# Dietary patterns and socioeconomic, demographic, and health-related behaviors during pregnancy. A cross-sectional study

**DOI:** 10.1590/1516-3180.2022.0629.R1.190523

**Published:** 2023-08-25

**Authors:** Tatiane Irene de Oliveira, Lais dos Santos, Doroteia Aparecida Höfelmann

**Affiliations:** I MSc. Nutritionist, Department of Nutrition, Postgraduate Program in Food and Nutrition (PPGAN), Universidade Federal do Paraná (UFPR), Curitiba (PR), Brazil.; II Nutricionist, Masters’ Student, Postgraduate Program in Collective Health, Departament of Public Health, Universidade Federal do Paraná (UFPR), Curitiba, Brazil.; III MSc, PhD. Nutritionist and Associate Professor, Department of Nutrition, Postgraduate Program in Food and Nutrition (PPGAN), Universidade Federal do Paraná (UFPR), Curitiba (PR), Brazil.

**Keywords:** Diet, food and nutrition, Pregnant women, Factor analysis, statistical, Feeding behavior, Feeding patterns, Food consumption, Food in pregnancy

## Abstract

**BACKGROUND:**

The identification and understanding of dietary factors and other characteristics that influence gestational weight gain can contribute to the formulation of strategies to promote healthy eating habits before and during pregnancy.

**OBJECTIVE:**

To investigate the association between dietary patterns, sociodemographic and obstetric characteristics, and health-related behaviors in pregnant women.

**DESIGN AND SETTING:**

A cross-sectional study was conducted on women undergoing prenatal care in the Unified Health System of Colombo, Paraná, Brazil, from February 2018 to September 2019.

**METHOD:**

A weekly food frequency questionnaire was administered, and dietary patterns were identified through factor analysis. Median regression models were constructed to identify the associations between dietary pattern scores and variables.

**RESULTS:**

Complete data were obtained from 495 pregnant women. Three dietary patterns were identified: 1) “healthy,” with higher factor loadings for the weekly consumption of raw vegetables, cooked vegetables, and fresh fruits; 2) “Western,” including soft drinks or artificial juice, candies, milk, and dairy products, and processed cold meat; and 3) “traditional,” beans and meat. Pregnant women aged 30 years or older (coefficient [Coef.] 0.86, 95% confidence interval [CI] 0.38–1.33) with moderate/intense physical activity (Coef. 0.32, 95% CI 0.02–0.62) had higher adherence to the “healthy” pattern. Adolescents and smokers adhered more to the “traditional” pattern (Coef. 0.17, 95% CI 0.01–0.33).

**CONCLUSION:**

Age, smoking status, and physical activity were associated with dietary patterns in pregnant women.

## INTRODUCTION

Eating habits during pregnancy are recognized as one of the environmental factors that can impact maternal health, and influence the development and long-term health of the fetus and child.^1^ Dietary restrictions are associated with nutritional deficiencies and low birth weight,^2,3^ while excessive gestational weight gain is associated with fetal macrosomia.^4,5^ These conditions are considered risk factors for the development of obesity and chronic diseases in adulthood.^2–5^

Data from the “Mamma & Bambino” cohort in Italy, comprising pregnant women, demonstrated that adherence to a healthier eating pattern was associated with a reduction in pre-gestational body mass index. Conversely, adherence to a less healthy eating pattern was associated with increased gestational weight gain, particularly among obese women.^6^

Analyzing dietary patterns is crucial for determining the associations between diet and diseases, as foods are consumed in complex combinations with interactions and synergies among their constituents.^7^ Therefore, the study of dietary patterns offers a broader view of food and nutrient consumption and overcomes methodological limitations related to previous studies that sought associations between nutrients or foods separately.^7,8^

Sociodemographic factors, health-related behaviors, food availability, and access influence dietary patterns, which can be characterized either by *a priori* knowledge (hypothesis-oriented approach) or *a posteriori* knowledge using data-driven techniques (empirically derived food patterns).^8,9,10,11,12,13^

Socioeconomic inequalities, demographic factors, and regional disparities can impact food access, quality, and the type of dietary pattern followed.^14,15^ Studies conducted in different locations have identified healthier eating patterns in groups with better social conditions, measured by education, income,^16–19^ and European origin.^20^ A study involving Italian and Mexican women found that a less healthy eating pattern was more prevalent among younger women.^15^ Another study conducted in Italy showed that women who engaged in higher levels of physical activity had a higher intake of vegetables.^21^

Conversely, populations with lower income diversity, unstable employment,^22^ and residing in peripheral areas of large cities may be more susceptible to adopting less healthy eating patterns.^12^ Furthermore, research on Brazilian pregnant women during prenatal follow-up in primary care showed that higher education was associated with a diverse and healthy eating pattern.^18,23^

Recent data from the Food and Nutrition Surveillance System in 2020 revealed that most pregnant women receiving prenatal follow-ups in the Brazilian Unified Health System (SUS) consumed fresh and minimally processed foods, such as fruits (76%) and vegetables (74%), indicating a healthy eating pattern. However, a substantial number also reported consuming sweetened beverages (56%) and ultra-processed foods (76%).^14^

In Brazil, nutritional guidelines and weight gain monitoring during the gestational period are common practices in prenatal care.^24^ However, identifying factors associated with adherence to specific dietary patterns can support health interventions and programs according to the social and economic profiles of pregnant women. This can improve the guidance and nutritional monitoring provided during prenatal care.^18^

## OBJECTIVE

The objective of this study was to identify dietary patterns and examine their association with demographic, socioeconomic, obstetric, and health-related behavioral factors in pregnant women receiving prenatal care.

## METHODS

This was a cross-sectional analysis of the “Study of the living and health conditions of pregnant and postpartum women.” The participants were pregnant women who received prenatal care at the SUS in the city of Colombo, Paraná, from February 2018 to September 2019.

Colombo is in the metropolitan region of the state capital of Paraná and was separated from the capital on February 5, 1890. It has a territorial area of 197,805 km^2^, with 95.1% of the households in urban areas and 85.0% having four or fewer residents in the household. In 2018, the Brazilian Institute of Geography and Statistics estimated the population to be 240,840 inhabitants.^25^

In 2017, the city had 18 (75%) Family Health Units, 5 (20.8%) Basic Health Units, and 1 (4.2%) Specialized Care Unit called Women’s Health Unit. The research included pregnant women at all levels of prenatal care (habitual, moderate, and high-risk) who received prenatal care in the SUS in Colombo, except for the maternity hospital in the municipality.

### Sample description

Based on the number of records in the Pregnancy Monitoring System (Sisprenatal) in Colombo in 2016 (3,807), sample calculations were conducted, considering a prevalence of 50% for the outcome, a margin of error of four percentage points, and a confidence level of 95%. A total of 520 pregnant women were scheduled for evaluation. Accounting for an estimated loss and refusal rate of 30% in longitudinal studies, 676 pregnant women were invited to participate in the study. During the consolidation of the fieldwork, incomplete questionnaires were identified for some variables. Thus, to enhance the statistical power of the study, another 59 pregnant women were selected to participate (n = 735). Estimates were generated using OpenEpi version 3.01, a free and open-source software (Georgia, United States).

The sample was distributed proportionally based on the number of registered pregnant women in each health unit, and all pregnant women were consecutively invited from the prenatal appointment agenda. The following inclusion criteria were adopted: pregnancy and prenatal care within the SUS in that city.

### Data collection

A questionnaire was administered to all the participants. Undergraduate nutrition students and nutritionists participated in data collection as interviewers. All interviewers received training, and the data collection instrument was pre-tested with different audiences at one of the municipal health units. A pilot study was conducted, covering all stages of the research, and participants involved in the pre-test, instrument test, and pilot study phases were excluded from the final sample.

### Quality control

Quality control measures were implemented for 12.6% (n = 70) of the questionnaires through telephone contact with the participants. A reduced version of the questionnaire consisting of four questions (full name, age, education, and full address of the participants) was used. Minor differences were observed in the study duration (1-year difference in three cases). However, the other information obtained during the interviews coincided in both instances.

### Questionnaires

The following variables were investigated: demographics – age (< 20, 20–29, and ≥ 30) and marital status (living with a partner or not); socioeconomic – education (0–7, 8–10 and ≥ 11 years of study) and paid activity (yes or no); obstetrics – gestational period (1^st^ trimester [0–13 weeks], 2^nd^ [14–26 weeks], and 3^rd^ [27 weeks or more]), and the number of pregnancies (1, 2, ≥ 3); health-related behaviors – level of physical activity (sedentary, mild, and moderate or intense) determined through the Physical Activity Questionnaire for Pregnant Women,^26,27^ alcohol consumption in the previous 12 months (yes or no), and current smoking status (yes or no) at the time of the survey. The option “ex-smoker” was included in the questionnaire, but was recategorized as current non-smoker in the analyses.

Furthermore, food consumption was assessed using a questionnaire based on the VIGITEL Surveillance System for Risk and Protection Factors for Chronic Diseases by Telephone Survey.^28^ This system, implemented by the Brazilian Ministry of Health since 2006, conducts surveys using electronic interaction to monitor the frequency and distribution of the main determinants of non-communicable chronic diseases in a probabilistic sample of adults. The VIGITEL questionnaire includes a validated section on food consumption,^29^ which addresses the weekly frequency of 12 food items: beans; at least one type of vegetable; salad or raw vegetable; cooked vegetable; red meat; chicken; fresh fruit; natural juice; soda or artificial juice; sweets, candies, desserts, chocolate, ice cream; milk, cheese, yogurt, and processed cold meat. The participants reported the weekly frequency of consumption for each item on a scale ranging from 0 (rarely) to 7 (daily, including weekends).

### Statistical analysis

For the descriptive analysis of the categorical variables, absolute frequencies (n) and relative frequencies (%) were reported. Continuous variables were presented using the mean and standard deviation. A list-wise deletion procedure was used for data analysis, which involved excluding pregnant women with incomplete data for any of the variables studied.

Dietary patterns were identified based on the information regarding the weekly frequency of food consumption. Factor analysis was conducted using the main factor extraction method with varimax orthogonal rotation. The adequacy of the sample to perform the factor analysis was evaluated using the Kaiser-Meier-Olkin test, yielding a global value of 0.72, indicating that the sample was adequate for the analysis.^30^ The number of factors to be retained was determined based on the factor loadings, eigenvalues, scree test, and interpretability of the generated factors. Internal consistency of the identified patterns was tested using Cronbach’s alpha. The adherence scores for each dietary pattern were derived from the regression method, employing the prediction command after the factorial analysis.

Quantile regression models were used to estimate the medians and their standard errors. These models were built using 100 bootstrap replications to identify the association between the resulting dietary patterns and the exposure variables. The quantile regression was employed to accommodate the non-normal distribution of the generated factors, which was confirmed by both the Shapiro-Wilk test and visual inspection of the factor score graphs.

In the adjusted analysis model, the variables were entered in the following order: 1) demographic and socioeconomic, 2) obstetric, and 3) health-related behaviors. The criterion for variable inclusion in the model was set at P < 0.20 for any of the categories. Statistical significance was set at P < 0.05. The regression results were expressed as coefficients (beta) with their corresponding 95% confidence intervals (CI).

### Ethical aspects

This research was approved by the Human Research Ethics Committee of the Health Sciences Sector of the Universidade Federal do Paraná (UFPR), (number 2405347) on November 29, 2017, and was conducted in accordance with the ethical standards required by the committee. All pregnant women who participated in the study signed the Free and Informed Consent Form (FICF). For participants under 18 years of age, both the participant and their legal guardian signed the FICF.

## RESULTS

Out of the 735 pregnant women invited to participate in the research, 604 agreed (82.3%). The mean age of the participating women was 26.0 years (95% CI 25.5–26.4). Of the 604 participants, 495 provided complete responses to the questionnaire and were included in this study. There were no considerable differences in terms of age or education between pregnant women with complete and incomplete data (data not shown).

Most pregnant women lived with a partner (79.6%), and 43.0% had 11 years of education or more, whereas 60.4% had no paid activity. Additionally, 42.4% of the participants were primiparous, and 49.9% were in the third trimester of pregnancy. In terms of health-related behaviors, 49.3% reported light activity levels, 7.5% were smokers, and 23.8% had consumed alcohol in the previous 12 months (Table 1).

**Table 1.** Distribution of demographic, socioeconomic, obstetric, and health-related behavioral factors of pregnant women in prenatal follow-up in the unified health system in Colombo, Paraná, 2018-2019. (n = 495)

Variable	n	%
**Demographic**		
***Age group***		
< 20 years	80	16.2
20–29 years	309	62.4
≥ 30 years	106	21.4
***Living with the partner***		
Yes	394	79.6
No	101	20.4
**Socioeconomic**		
***Education***		
0–7 years	90	18.2
8–10 years	192	38.8
≥11 years	213	43.0
***Paid activity***		
No	299	60.4
Yes	196	39.6
**Obstetrics**		
***Gestational period***		
1^st^ trimester	75	15.2
2^nd^ trimester	173	34.9
3^rd^ trimester	247	49.9
***Number of pregnancies***		
1^st^ pregnancy	210	42.4
2^nd^ pregnancy	156	31.5
3^rd^ pregnancy	129	26.1
**Health-related behavior**		
***Physical activity***		
Sedentary	54	10.9
Light	244	49.3
Moderate/Intense	197	39.8
***Current smoking***		
No	458	92.5
Yes	37	7.5
***Alcohol***		
No	377	76.2
Yes	118	23.8

**Figure 1.**
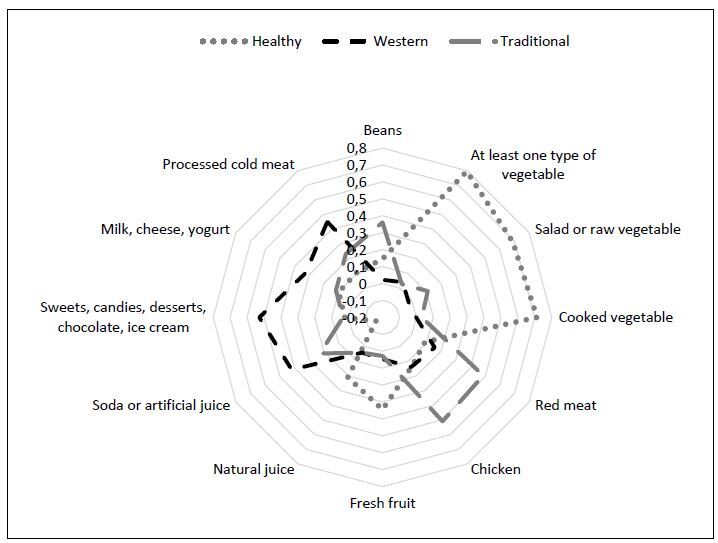
Factorial loads of dietary patterns according to weekly dietary frequency of pregnant women in prenatal follow-up. Colombo, Paraná, 2018-2019 (n = 495).

The following three dietary patterns were identified: “healthy,” “Western,” and “traditional” diets. 1) The “healthy” pattern exhibited the highest factor loadings for weekly consumption of at least one type of vegetable (0.79), salad or raw vegetables (0.68), cooked vegetables (0.72), and fresh fruit (0.34); 2) The “Western” pattern was characterized by weekly consumption of soft drinks or artificial juice (0.42), sweets (candies, dessert, chocolate, ice cream) (0.54), milk and dairy products (0.32), and processed cold meat (0. 44); 3) The “traditional” pattern was characterized by weekly consumption of beans (0.35), red meat (0.47), and chicken (0.49). Among the 12 food items, only the natural juice had a factor loading below 0.30 (Figure1). The first factor explained 64.8% of the data variability, the second explained 28.9%, and the third explained 24.9% of the variability. The Cronbach’s alpha values for the identified patterns were 0.76, 0.54, and 0.50, respectively.

The associations between the three identified dietary patterns and demographic, socioeconomic, obstetric, and health-related behavioral variables are presented in Tables 2, 3, and 4.

**Table 2.** Coefficients of the quantile regression of the “healthy” dietary standard of pregnant women, according to demographic, socioeconomic, obstetric, and health-related behavioral factors. Colombo, Paraná, 2018-2019 (n = 495)

Variables Demographic	Percentile 50					
	Not adjusted			Adjusted		
	Coef	CI 95%	P*	Coef	CI 95%	P
***Age group***			**< 0.001**			**0.002** ^**a**^
< 20 years	Ref.			Ref.		
20–29 years	0.36	0.07;0.65	**0.013**	0.31	-0.00;0.63	0.051^a^
≥ 30 years	0.94	0.50;1.37	**< 0.001**	0.86	0.38;1.33	**< 0.001** ^**a**^
***Living with the partner***			0.174			0.566^a^
Yes	Ref.					
No	-0.19	-0.47;0.08		-0.09	-0.41;0.21	
**Socioeconomic**						
***Education***			**0.003**			0.054^b^
0–7 years	Ref.					
8–10 years	0.09	-0.23;0.42	0.569	0.08	-0.32;0.49	0.695^b^
≥ 11 years	0.33	0.05;0.61	**0.018**	0.28	-0.07;0.63	0.116^b^
***Paid activity***			**0.001**			0.107^b^
No	Ref.					
Yes	0.35	0.14;0.56		0.13	-0.07;0.34	
**Obstetrics**						
***Gestational period***	Ref.		0.565			
1^st^ trimester	0.07	-0.22;0.37	0.633			
2^nd^ trimester	-0.06	-0.37;0.25	0.708			
3^rd^ trimester						
***Number of pregnancies***			0.217			
1^st^ pregnancy	Ref.					0.923^c^
2^nd^ pregnancy	0.39	0.04;0.74	**0.028**	0.15	-0.15;0.46	0.321^c^
3^rd^ pregnancy	0.15	-0.13;0.44	0.297	-0.02	-0.36;0.31	0.884^c^
**Health-related behaviors**						
***Physical activity***			**0.003**			0.053^d^
Sedentary	Ref.			Ref.		
Light	0.33	0.06;0.59	**0.014**	0.18	-0.10;0.48	0.209^d^
Moderate/Intense	0.51	0.25;0.77	**< 0.001**	0.32	0.02;0.62	**0.036** ^**d**^
***Current smoking***			0.115			0.103^d^
No	Ref.			Ref.		
Yes	0.44	-0.11;1.00	0.115	0.40	-0.08;0.89	
***Alcohol***			0.595			
No	Ref.					
Yes	-0.07	-0.33;0.19	0.595			

Coef = coefficient (beta); CI = Confidence interval; Ref = reference category. *Median regression with 100 bootstrap replications; ^a^Adjusted for demographic variables; ^b^Adjusted for demographic and socioeconomic variables; ^c^Adjusted for demographic, socioeconomic, and obstetric variables; ^d^Adjusted for demographic, socioeconomic, obstetrics, and health-related behavioral variables.

**Table 3.** Coefficients of the quantile regression of the “Western” dietary standard of pregnant women, according to demographic, socioeconomic, obstetric, and health- related behavioral characteristics. Colombo, Paraná, 2018-2019 (n = 495)

Variables Demographic	Percentile 50					
	Not adjusted			Adjusted		
	Coef	CI 95%	P*	Coef	CI 95%	P
***Age group***			0.082			0.095^a^
< 20 years	Ref.			Ref.		
20–29 years	-0.00	-0.35; 0.35	0.993	-0.00	-0.32; 0.32	0.992^a^
≥ 30 years	-0.23	-0.63; 0.16	0.253	-0.23	-0.58; 0.11	0.185^a^
***Living with the partner***			0.355			
Yes	Ref.					
No	0.09	-0.10; 0.30				
**Socioeconomic**						
***Education***			0.650			
0–7 years	Ref.					
8–10 years	0.05	-0.27; 0.38	0.732			
≥11 years	0.07	-0.26; 0.42	0.646			
***Paid activity***			0.790			
No	Ref.					
Yes	0.02	-0.13; 0.17				
**Obstetrics**						
***Gestational period***			0.783			
1^st^ trimester	Ref.					
2^nd^ trimester	-0.05	-0.25; 0.13	0.548			
3^rd^ trimester	-0.03	-0.26; 0.18	0.745			
***Number of pregnancies***			0.643			
1^st^ pregnancy	Ref.					
2^nd^ pregnancy	-0.13	-0.23; 0.19	0.899			
3^rd^ pregnancy	0.07	-0.10; 0.25	0.414			
**Health-related behaviors**						
***Physical activity***			0.169			0.127^b^
Sedentary	Ref.			Ref.		
Light	0.22	-0.03; 0.47	0.084	0.21	-0.04; 0.47	0.110^b^
Moderate/Intense	0.27	0.02; 0.56	0.070	0.24	-0.07; 0.57	0.138^b^
***Current smoking***			0.309			
No	Ref.					
Yes	0.16	-0.15; 0.49				
***Alcohol***			**0.041**			0.220^b^
No	Ref.			Ref.		
Yes	0.17	0.01; 0.33		0.19	-0.34; 0.50	

Coef = coefficient (beta); CI = confidence interval; Ref = reference category. *Median regression with 100 bootstrap replications; ^a^Adjusted for demographic variables; ^b^Adjusted for demographic, obstetric, and health-related behavioral variables.

**Table 4.** Coefficients of the quantile regression of the “traditional” dietary pattern of pregnant women, according to demographic, socioeconomic, obstetric, and behavioral characteristics. Colombo, Paraná, 2018-2019 (n = 495)

Variables Demographic	Percentile 50					
	Not adjusted			Adjusted		
	Coef	CI 95%	P*	Coef	CI 95%	P
***Age group***			**0.043**			0.037^a^
< 20 years	Ref.			Ref.		
20–29 years	-0.01	-0.25; 0.23	0.927	-0.01	-0.25; 0.23	0.926^a^
≥ 30 years	-0.21	-0.46; 0.03	0.088	-0.21	-0.46; 0.02	0.083^a^
***Living with the partner***			0.740			
Yes	Ref.					
No	-0.03	-0.23; 0.16	0.740			
**Socioeconomic**		**-0.18; 0.31**	**0.629**			
***Education***	.		0.539			
0–7 years	Ref.					
8–10 years	0.20	-0.02; 0.42	0.085			
≥ 11 years	0.12	0.08; 0.34	0.234			
***Paid activity***			0.257			0.260^b^
No	Ref.			Ref.		
Yes	-0.08	-0.22; 0.05		-0.06	0.19; 0.05	
**Obstetrics**						
***Gestational period***			0.178			0.476^c^
1^st^ trimester	Ref.			Ref.		
2^nd^ trimester	-0.21	-0.43; 0.00	0.053	-0.19	-0.40; 0.02	0.081^c^
3^rd^ trimester	-0.23	-0.45; -0.01	**0.037**	-0.15	-0.38; 0.08	0.203^c^
***Number of pregnancies***			0.988			
1^st^ pregnancy	Ref.					
2^nd^ pregnancy	0.07	-0.12; 0.28	0.448			
3^rd^ pregnancy	-0.01	-0.18; 0.15	0.878			
**Health-related behaviors**						
***Physical activity***			0.124			0.112^d^
Sedentary	Ref.			Ref.		
Light	0.07	-0.24; 0.38	0.659	0.01	-0.30; 0.32	0.950^d^
Moderate/intense	0.17	-0.16; 0.50	0.320	0.12	-0.20; 0.44	0.461^d^
***Current smoking***			**0.042**			**0.041** ^**d**^
No	Ref.			Ref.		
Yes	0.16	0.01; 0.31		0.17	0.01; 0.33	
***Alcohol***			0.509			
No	Ref.					
Yes	0.05	-0.11; 0.23				

Coef = coefficient (beta); CI = confidence interval; Ref = reference Category. *Median regression with 100 bootstrap replications; ^a^Adjusted for demographic variables; ^b^Adjusted for demographic and socioeconomic variables; ^c^Adjusted for demographic, socioeconomic, and obstetric variables; ^d^Adjusted for demographic, socioeconomic, obstetric, and health-related behavioral variables.

It was found that pregnant women aged 30 years or older had higher adherence scores to the “healthy” dietary pattern (Coef. 0.94, 95% CI 0.50–1.37), as well as those with 11 years or more of education (Coef. 0.33, 95% CI 0.05–0.61). Pregnant women who engaged in paid activities also had higher scores in this pattern (Coef. 0.35, 95% CI 0.14–0.56), along with those in their second pregnancy (Coef. 0.39, 95% CI 0.04–0.74) and those with a level of moderate/intense physical activity (Coef. 0.51, 95% CI 0.25–0.77). After adjusting for the variables, pregnant women aged 30 years or older still exhibited higher scores in this pattern (Coef. 0.86, 95% CI 0.38–1.33), as well as those with moderate or intense physical activity (Coef. 0.32, 95% CI 0.02–0.62) (Table 2).

Pregnant women who reported alcohol consumption in the previous 12 months had higher adherence scores for the “Western” dietary pattern (Coef. 0.17, 95% CI 0.01–0.33). However, after adjusting for other model variables, the association was no longer significant (Table 3).

A lower adherence to the “traditional” pattern was observed with advancing age (P = 0.043) and in the third trimester of pregnancy (Coef. -0.23, 95% CI -0.45–-0.01). However, pregnant smokers (Coef. 0.16, 95% CI 0.01–0.31) had higher adherence scores for this pattern. After adjusting for the other variables, the association between age and smoking remained significant for the “traditional” pattern (Table 4).

## DISCUSSION

Three dietary patterns were identified among the evaluated pregnant women: “healthy,” characterized by the higher consumption of vegetables and fresh fruits; “Western,” with greater consumption of soft drinks or artificial juice, sweets, milk, and dairy products, and processed cold meat; and “traditional”, characterized by higher consumption of beans, red meat, and chicken.

These dietary patterns are similar to those identified in other studies with Brazilian pregnant women^31,32,33,34,35^ and in studies from other countries, such as the Norwegian cohort of mothers and children,^36^ which comprised 66,000 women from 2002 to 2008, and the Italian study of *Mamma & Bambino*.^9^ These patterns are similar not only in terms of food groups but also in their type or nature: one is characterized by the presence of foods such as fruits and vegetables; another is characterized by the consumption of ready-to-eat and/or high-energy products; and the third includes more traditional foods in the diet of a certain region or country.

Adherence to the “healthy” pattern in this study was higher among women aged 30 years or older, those who lived with a partner, had a paid job, and had a higher level of education, suggesting a more stable socioeconomic condition and a higher socioeconomic level. These findings agree with a study conducted in Australia that investigated changes in the dietary patterns of 621 women from preconception to pregnancy. Moreover, it identified that higher education and higher income were associated with healthy eating patterns.^17^ Similarly, in the Conditions Affecting Neurocognitive Development and Learning in Early childhood study conducted in the United States from 2006 to 2011, the “healthy” pattern was associated with pregnant women who were on average 30 years old and with higher levels of education.^37^

Additionally, age and education were positively associated with the “healthy” dietary pattern in a sample of low-income American women. The objective of their study was to identify factors that influenced diet quality.^38^ Evidence consistently points to a social gradient, where women with higher age, education, and income, or other indicators of a better socioeconomic status tend to have healthier eating patterns. These factors play a crucial role in the consumption of healthier foods as they provide greater capacity to purchase food, access to places that sell higher quality products, and the access to adequate information.^39^ The data from the Household Budget Survey conducted in Brazil between 2017 and 2018 revealed that the purchase of fruits and vegetables was lower among families with lower incomes.^40^

A prospective study in the cities of Petrópolis and Queimados, Rio de Janeiro, from 2007 to 2008,^31^ identified higher adherence to the “Western” dietary pattern, composed of foods with high energy density and low nutritional value, among pregnant women from economic classes B or C and with higher parity. However, in the present study, no association was found between education, parity, and the “Western” dietary pattern. However, there was an association between this pattern and alcohol consumption, but the association was no longer significant after adjusting for other variables in the model.

Research has shown that exercise during pregnancy has a positive impact on the health of both mother and child.^41,42^ In the present study, a moderate and/or intense level of physical activity was associated with higher adherence scores for the “healthy” pattern; likewise, other studies concluded that pregnant women who engage in physical activity during their leisure time made healthier food choices.^43,44,45,46^ Furthermore, a systematic review that evaluated the effect of physical exercise on taste perception identified that regular physical activity has significant effects on taste intensity. By exercising frequently, individuals can experience faster satiety, reduce the consumption of highly flavorful and energetic foods, and decrease their overall food intake, leading to healthier food choices.^47^

Smokers and younger pregnant women demonstrated higher adherence scores to the “traditional” pattern in this study, which included the consumption of chicken, red meat, and beans. Similar findings were observed in the Brazilian cohort between 2007 and 2008 in Rio de Janeiro, which investigated dietary patterns and their association with the baby’s birth weight. Moreover, younger pregnant women and smokers exhibited greater adherence to the “traditional” pattern, consisting of beans, rice, vegetables, bread, butter/margarine, and sugar, and to the prudent dietary pattern characterized by red meat and chicken.^36^

In a study conducted in the south of England, the food consumption of 12,053 pregnant women was evaluated, and the “traditional” dietary pattern, characterized by the consumption of red meat, poultry, and other foods, was more frequent among younger pregnant women.^48^ Moreover, the results of the study by Barchitta et al., demonstrated that pregnant women who smoke are more likely to have inadequate folate intake, which may be associated with a limited variety in their eating pattern.^49^

The food frequency questionnaire (FFQ), the method used to assess food consumption in the present study, is widely employed in epidemiological studies due to its fast application and efficiency in identifying the usual food consumption, in addition to its low-cost.^50^ However, it does have certain limitations. The FFQ evaluates food consumption qualitatively, without measuring the exact quantities consumed or providing detailed options for food items. In addition, the FFQ is subject to memory bias, the individual’s ability to recall the frequency and retrospectively quantify the foods consumed influencing the potential for underestimation or overestimation.^51^

Factor analysis, which is the extraction method used in this study, is widely used to explore dietary patterns. However, its application involves arbitrary decision-making, which includes the formation of food groups, pattern retention, and interpretation based on scientific knowledge about the studied population’s food and diet.^52^ Future studies could benefit from applying alternative approaches, such as the Ecological Momentary Assessment of Diet^53,54,55^ or Clustering on Principal Components,^56^ to address the technical limitations of the FFQ and factor analysis or principal component analysis in deriving dietary patterns.

Furthermore, the relative socioeconomic homogeneity of the sample, consisting mostly of pregnant women undergoing prenatal care at the SUS, may have influenced the obtained results. Although a probabilistic sampling procedure was not performed, the high number of pregnant women attending prenatal appointments, the repeated presence of researchers in the Health Units at different times, and the selection of pregnant women from all the health units contributed to enhancing the internal validity of the information.

It is also important to acknowledge the cross-sectional design of this analysis, which captures the characteristics of the moment in which the data were collected, preventing the establishment of causal relationships between the derived dietary patterns and the investigated exposures. This limitation should be noted. However, the study demonstrates methodological rigor, including double typing and sample quality control. Additionally, the diverse range of the characteristics evaluated allowed for a comprehensive assessment of the predictors of food consumption among pregnant women.

## CONCLUSIONS

Demographic, socioeconomic, and health-related behavioral factors were associated with the dietary patterns of pregnant women receiving prenatal care in the metropolitan region of Curitiba, Paraná. Specifically, age, the level of moderate/intense physical activity, and tobacco use were highlighted as important factors in this study.

The identification of these factors is crucial in prenatal care as it enables the establishment of effective health promotion interventions aimed at reducing the potential risks to maternal and child health.
